# Synthesis and Characterization of Imidazolium-Based Ionic Liquids and Evaluating Their Performance as Asphaltene Dispersants

**DOI:** 10.3390/ma15041600

**Published:** 2022-02-21

**Authors:** Alaa Ghanem, Rima D. Alharthy, Saad M. Desouky, Raghda A. El-Nagar

**Affiliations:** 1 PVT-Lab, Production Department, Egyptian Petroleum Research Institute, Nasr City, Cairo 11727, Egypt; usdesouky@yahoo.com; 2 Department of Chemistry, Science & Arts College, Rabigh Branch, King Abdulaziz University, Rabigh 21911, Saudi Arabia; 3 Oil Lab Analysis, Analysis & Evaluation Department, Egyptian Petroleum Research Institute, Nasr City, Cairo 11727, Egypt

**Keywords:** ionic liquids (ILs), asphaltene dispersants, onset precipitation, surface activity

## Abstract

With the projected increase in the production of heavy oil due to the energy crisis, asphaltene-related issues are likely to come to the forefront. This leads to operational problems, safety hazards, and oil production deficiencies, resulting in huge economic losses for the petroleum industry. Therefore, in this work, we aimed to inhibit asphaltene precipitation using ionic liquid (IL) compounds. ILs with long alkyl chains can inhibit the precipitation of asphaltene molecules due to the π–π* interactions between them and the formation of hydrogen bonds. A series of imidazolium-based ionic liquids, IL-0, IL-4, IL-10, and IL-16, were synthesized with yield percents of 79, 81, 80, and 83%, respectively. The prepared materials were characterized well using FTIR, ^1^H-NMR, and Elemental Analysis. The surface tension, interfacial tension (IFT), and different surface parameters were investigated at different temperatures to simulate the reservoir temperature. IL-0, IL-4, IL-10, and IL-16 displayed their γ_cmc_ values at 35, 34, 31, and 32 mN/m at 303 °K, respectively. It was found that the prepared ILs are good surfactants with low values of interfacial tension. Quantum structure–activity relationships using Density Functional Theory (DFT) were used to investigate the geometry optimization electronic structures, the energy gap (ΔE), and the reactivity of the cations of the prepared ILs. The synthesized ILs were evaluated as asphaltene dispersants using two different techniques. The viscometric technique showed that the asphaltene onset precipitation was 28.5 vol.%. This percent was postponed to 42.8, 50, 78.5, and 64.3 vol.%, after adding IL-0, IL-4, IL-10, and IL-16, respectively, and the spectroscopic technique confirmed the results.

## 1. Introduction

Crude oil is known as a complex mixture of different hydrocarbons, which are classified into many groups, including polar and nonpolar compounds, such as saturates, aromatics, resins, and asphaltenes. Waxes and asphaltenes stand out as the main causes of organic deposition through these substances [1,2]. Asphaltenes, as the heaviest and least understood crude oil components (poly-condensed aromatic rings, with alkyl radicals of various sizes and functional groups formed by oxygen, nitrogen, and sulfur, as well as heavy metals, such as vanadium, nickel, and iron, which have high structural complexity), are able to completely dissolve in aromatic solvents when deposited in a paraffinic solvent. Resins are not the same as asphaltenes in the electrical charges that attract asphaltene molecules [3,4,5,6,7,8]. Moreover, resins are connected to a larger number of alkyl chains, which increases the resins’ flowability in the oil. This relation results in the formation of what is known as a stable layer beyond the asphaltene structure, which acts as a barricade between the polar molecules of asphaltenes and the nonpolar ones in the oil [9,10,11,12], thus acting as natural inhibitors and asphaltene-stabilizing in crude oil [13,14,15,16,17]. The onset of precipitation occurs once the constituent asphaltene exceeds the critical value. Many factors influence the precipitation of asphaltene, including changes in oil pressure, temperature, or chemical composition caused by gas injection, mixing of different oil streams, or phase separation [18,19]. Asphaltenes exhibit a wide range of intermolecular interactions. For example, the aromatic nucleus exhibits π–π* interactions; acid–base interactions refer to a large number of different heteroatoms; the long alkyl chains are associated with each other, and functional groups, such as hydroxyl and carboxyl, interact with each other via hydrogen bonds; and metals can interact via charge transfer [20,21,22].

All of these interactions result in the phenomena of asphaltene self-association. Agglomeration and precipitation of asphaltene from crude oil have been identified as the most pressing problems during the production of oil recovery, transportation, and production, such as extraction and refining [23,24]. Asphaltene deposition can occur in various locations, such as formation near the wellbore, wellbore pumps, tubing, chokes, and flowlines, which causes a lot of problems during the production or transportation of oil [25]. In addition, the clogging or blocking of oil reservoir pores, as well as the alteration of the rock wettability, has a significant effect on the oil production. Furthermore, the movement of precipitated asphaltenes may cause problems with crude oil storage during the refinery processes [26]. As a result, asphaltene precipitation mitigation is a critical point in the petroleum industry to avoid exorbitant costs for installed asphaltene remedial solutions. However, the petroleum industry turned to extracting asphaltenic heavy crude oil because of the looming energy crisis, even though there are other alternatives, such as solar energy and syngas [27,28]. Therefore, several pieces of research have been conducted in the last few years to study the issues related to the deposited asphaltenes and to develop appropriate solutions [25,26]. Chemicals were designed to mimic the structure of resins to interact with the surface of asphaltene via adsorption, electrostatic attraction, or/and hydrogen bonding. This is an effective technique to peptize or dissolve asphaltene molecules in the crude oil mixture [29]. Alcohols [30], organic acids [31], vegetable oils [32], and amphiphiles [33] have all shown promising results in asphaltene precipitation [11,34,35,36,37,38,39,40]. Today, ionic liquids (ILs) attract great attention because they are environmentally friendly alternatives to organic solvents [41,42]. ILs possess unique physicochemical characteristics, such as high surface-active parameters, nontoxic, low vapor pressure, and are non-corrosive, non-flammable, and have high thermal stability. Ionic liquids are mainly composed of amphiphilic cation: pyridinium, ammonium, imidazolium, phosphonium or sulfonium cation and inorganic or organic anions, halides, phosphate, sulfate, or phosphonate [43,44,45,46]. The different applications of ILs in the petroleum field have been recognized, especially when using room temperature ionic liquids (RTILs) [29]. RTILs, such as triethylammonium acetate and triethylammonium phosphate-based ionic liquids, were used to improve the viscosity of heavy crude oil at upstream conditions. The results showed a significant improvement in viscosity reduction at the oil reservoir’s optimum temperature conditions, while imidazolium-based ionic liquids were investigated as asphaltene inhibitors [47]. Hu and Guo studied different ILs to reduce asphaltene precipitation and improve crude oil stability [48]. Fan et al. employed the synergistic impact of alkyl imidazolium-based ionic liquid on both the asphaltene content and the viscosity reduction of the crude oil [49]. Bowers et al. [50] studied the surface tension of alkyl imidazolium ionic liquids and determined the critical micellar concentration, which was similar to that of a short chain cationic surfactant. Nandwani et al. [51] studied the interfacial tension (IFT) of C_16_mimBr ionic liquid and demonstrated its superiority in lowering the IFT and releasing a higher amount of oil from the oil/water system when compared with the known cationic surfactant CTAB, in addition to being used efficiently under high temperature and high salinity [52].

In the present work, a series of alkyl imidazolium-based acidic ionic liquid (highly aromatic structures) are synthesized and characterized using FTIR, ^1^H NMR, TGA, and Elemental Analysis. In addition, the surface tension of the prepared compounds is investigated at different temperatures. Quantum structure–activity relationships using Density Functional Theory (DFT) are utilized to predict the impact of the molecular structures on the dispersion of asphaltene. The prepared ILs are evaluated as asphaltene dispersants by determining the onset precipitation point of asphaltene using both spectroscopic and viscometry methods.

## 2. Experimental Section

### 2.1. Materials

All of the chemicals and reagents used were of analytical grade and used directly without purification. Imidazole (≥99%), 1-chlorobutane (≥99%), 1-chlorodecane (≥98%), 1-chlorohexadecane (≥99%), potassium hydroxide (≥98%), dodecyl benzene sulfonic acid (DBSA) (≥99%), heptane (≥97%), n-hexane (≥99%), benzene (≥98%), and neutral aluminum oxide and chloroform (≥98%) were all supplied from Merck (Darmstadt, Germany). Heavy crude oil was delivered from GPC petroleum company, Cairo, Egypt. The physical properties and the group composition analysis (SARA) are listed in Table 1 and Table 2.

Table 1 displays a low value of API gravity and high values of density, kinematic viscosity, and asphaltene content. Table 2 demonstrates the SARA analysis of the heavy crude oil, which contains a low content of saturates and a high content of heavy aromatic hydrocarbons, such as asphaltene, resin, and aromatics.

### 2.2. Methodology

The synthesis of IL-0, IL-4, IL-10, and IL-16 is illustrated as follows:

A series of alkyl imidazoles were prepared by mixing imidazole (0.1 mol) with potassium hydroxide in 50 mL of acetonitrile with vigorous stirring. After the complete miscibility, 1-Chlorobutane, 1-Chlorodecane, or 1-Chlorohexadecane (0.1 mol) was added, drop by drop, to the previous mixture for three hours until a white precipitate (KCl) was noticed. After, it was removed by filtration, while the filtrate was concentrated under vacuum to obtain different alky imidazoles [53].

**Scheme 1:**
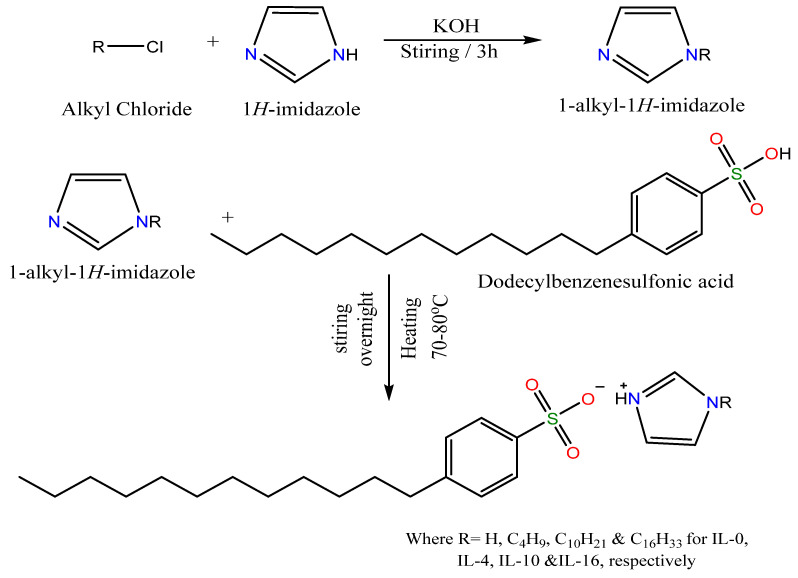
Synthesis mechanism of IL-0, IL-4, IL-10, and IL-16.

1-alkyl imidazole derivatives were refluxed and stirred overnight with DBSA at 70–80 °C (Scheme 1) [2]. The purity of the synthesized ILs was confirmed using the TLC technique, which indicated that they possessed good solubility in different polar solvents.

### 2.3. Characterization of the Prepared ILs

The characterization of the four ILs was obtained by FTIR spectra, which was recorded in the range of 400–4000 cm^−1^ using a Nicolet Ia-10 spectrometer(Thermo Fisher Scientific, Waltham, MA, USA). ^1^H NMR spectra of the ILs were identified at room temperature on BRUKER ^1^H-NMR spectroscopy (Billerica, MA, USA) at 400 MHZ using D_2_O solvent. Then, for better elucidation, the peaks were identified on MestreNova software (Trial version, Mestrelab Research, Santiago de Compostela, A Coruña, Spain) [2]. Thermal gravimetric analyses (TGA) were conducted on Thermal Analyzer SDT Q500 V20.10 Build 36 (Mettler Toledo, Greifensee, Switzerland) with a heating rate of 10 °C/min. The values of both surface and interfacial tension of the ILs solutions were determined at different temperatures (303, 313, and 323 K) using a Du Nouy tensiometer with a platinum ring (KRUSS, Hamburg, Germany). The instrument was first calibrated using deionized water, which is around 72 ± 0.5 mN/m [54]. Then, different concentrations (in the range of 0.001–0.00001 mol/L) of aqueous solutions, containing the prepared ILs, were measured.

### 2.4. Asphaltene Extraction Method

The asphaltene utilized in this study was extracted from the heavy crude oil of the GPC according to the standard method IP-143. A total of 10 g of the GPC crude oil was refluxed with 300 mL n-heptane for one hour. Then, the mixture was cooled in a dark cupboard for two hours. After, the mixture was filtered using filter paper. The filter paper containing the precipitate (the precipitate contains asphaltene, waxy substances, and inorganic materials) was folded and placed in an extractor to be washed with hot n-heptane to remove any waxy substances. Then, the asphaltene was separated from the inorganic materials by the dissolution in hot toluene. Finally, the solvent was evaporated, and the asphaltene was weighted to calculate its content from the equation

A = 100 ∗ (M/G),

where M is the mass of asphaltene in g, and G is the mass of crude oil.

### 2.5. Determining the Onset of Asphaltene Precipitation Using the Viscometric Method

The viscometry technique [2] is generally utilized to investigate the onset precipitation point of asphaltene. In this method, the asphaltenic crude oil was titrated with a series of consecutive volumes of a precipitant, such as n-heptane. For each run, series of samples were prepared to cover the solvent concentration range of 0–100 vol percent. Then, the kinematic viscosity of the samples was determined by Stabinger viscometer with uncertainty equal to 0.3 cSt. (SVM 3001 Anton Paar, Graz, Austria) at 40 °C. The experiment was repeated by adding each IL at different concentrations to reach the optimum concentration.

### 2.6. Determining the Onset of Asphaltene Precipitation Using the UV Spectroscopic Method

**Scheme 2:**
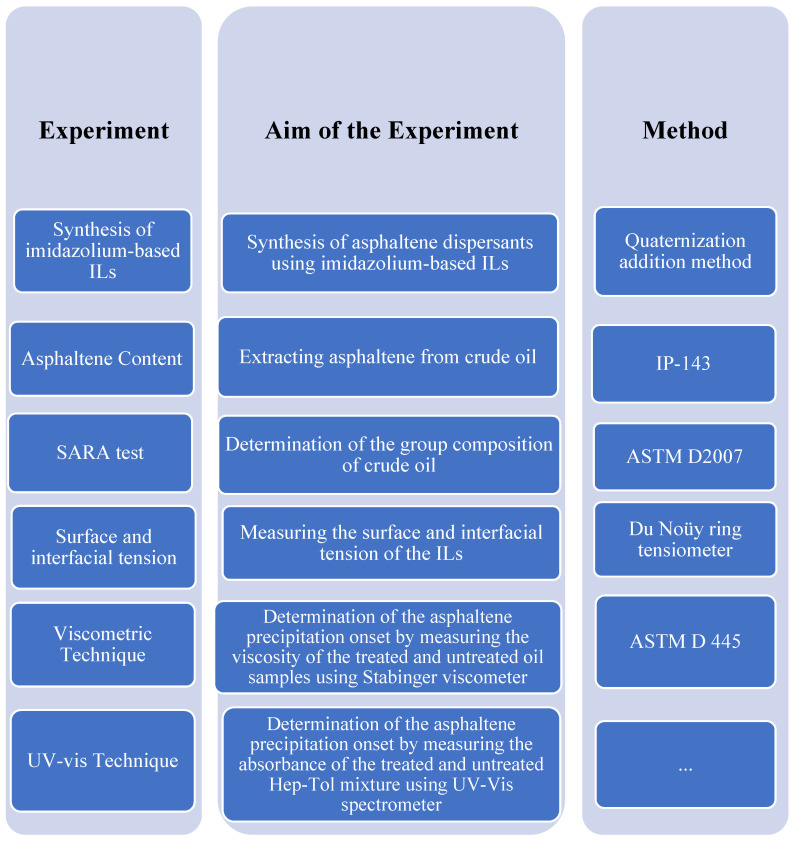
Flowchart includes the aim of the main experiments in this research.

A total of 0.1 g of the extracted asphaltene was added to 100 mL of toluene to prepare a stock solution of 1000 ppm. Then, a mixture of n-Heptane/toluene (Hep-Tol mixture) in the range of 0/100, 10/90, 20/80, 30/70, 40/60, 50/50, 60/ 40, 70/30, 80/20, 90/10, and 100/0 mL/mL was prepared in 15 mL centrifuge bottles. A known portion of the asphaltene stock solution (50 ppm) was added to each bottle. The samples were shaken for 5 min and left standing for 24 h. Next, the prepared samples were centrifuged at 5000 rpm for 15 min. Then, only 3 mL from the top of each sample was decanted and used to measure the absorbance via a JASCO V-750 UV-vis spectrometer (JASCO, Tokyo, Japan) in the wavelength range of 190–900 nm. The previous step was repeated in the presence of the prepared ILs to investigate their effect [55]. By increasing the amount of n-heptane, the optical density gradually decreased until a sudden decrease was observed, which represents the onset of precipitation. Scheme 2 displays a flowchart that summarizes the main experiments and the aim of each experiment in this research.

## 3. Results and Discussion

### 3.1. Description of the Synthesized Ionic Liquids

The molecular structure of the prepared ILs, the molecular weight, and the yield of each IL are presented in Table 3. The molecular weight of IL-0, IL-4, IL-10, and IL-16 is 394.5, 450.6, 534.8, and 619, respectively, while the yield ranged between 79 and 83%.

The chemical structures were confirmed via the following different tools of analysis.

### 3.2. Elemental Analysis

The content of the elements in the prepared ILs provides information regarding the role of the organic molecules, where the molecular formula can be confirmed by comparing the theoretical and the experimental data. The data in Table 4 show that the calculated values of C, H, N, O, and S are compatible with the observed values.

### 3.3. FT-IR Spectra

**Figure 1:**
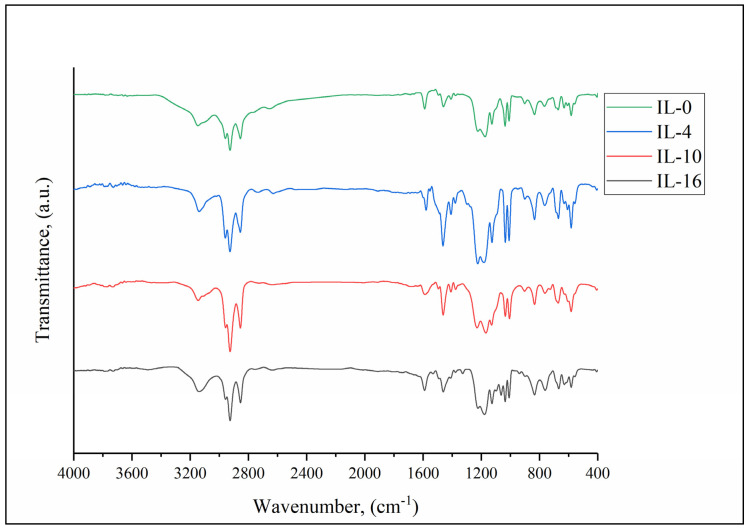
FT-IR spectra of IL-0, IL-4, IL-10, and IL-16.

(Figure 1) of IL-0, IL-4, IL-10, and IL-16 were investigated, and the characteristic bands are summarized in Table 5, which confirmed the chemical structures of the newly prepared ILs [56]. Aromatic C-H stretching bands appeared between 3137 and 3147 cm^−1^, while aliphatic stretching C-H bands appeared around 2854 and 2960 cm^−1^. At the same time, C-C vibrations in the imidazole ring appeared between 1579 and 1589 cm^−1^. Stretching bands between 1460 and 1462 cm^−1^ are related to aromatic C=C. The stretching bands of C-N (vibration modes) in the imidazole ring were found in the range of 1407–1409 cm^−1^. The stretching vibration peaks at 1372–1377 cm^−1^ are assigned to S=O. Symmetric stretching of the SO3^−^ group was recorded at 1034–1036 cm^−^^1^.

### 3.4. ^1^H NMR Spectra

**Figure 2:**
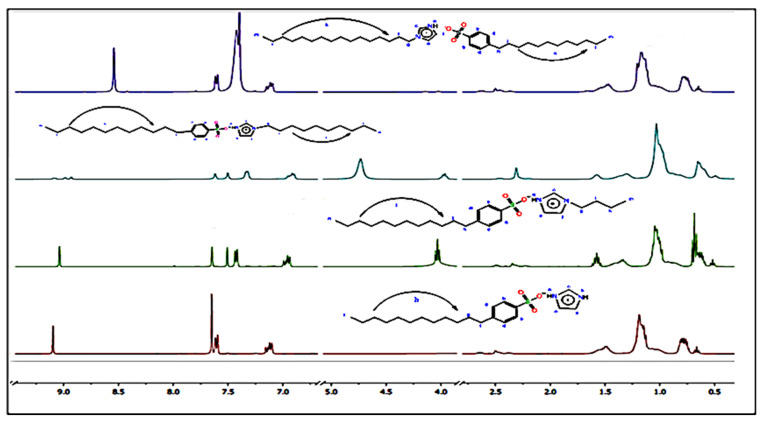
^1^H NMR spectra of IL-0, IL-4, IL-10, and IL-16.

1H NMR spectra of IL-0, IL-4, IL-10, and IL-16 are presented in Figure 2 and Table 6. The highly de-shielded protons a, c, and e, for IL-0, and a, c, e, and f, for IL-4, IL-10, and IL-16, have a high δ value due to the withdrawing action of N in the imidazolium ring. Protons b and d are not similar, whereas b is affected directly by the SO_3_^−^ group. The methyl protons f, g, and h, for IL-0, and h, I, j, k, and l, for IL-4, IL-10, and IL-16, are highly de-shielded due to the fact that they are attached directly to the aromatic ring, and are electron-withdrawing groups. The aliphatic methyl protons I, for IL-0; n, for IL-4; and m, for IL-10 and IL-16, appeared in triplet at the lowest δ value.

### 3.5. Thermal Gravimetric Analysis of the Prepared ILs

**Figure 3:**
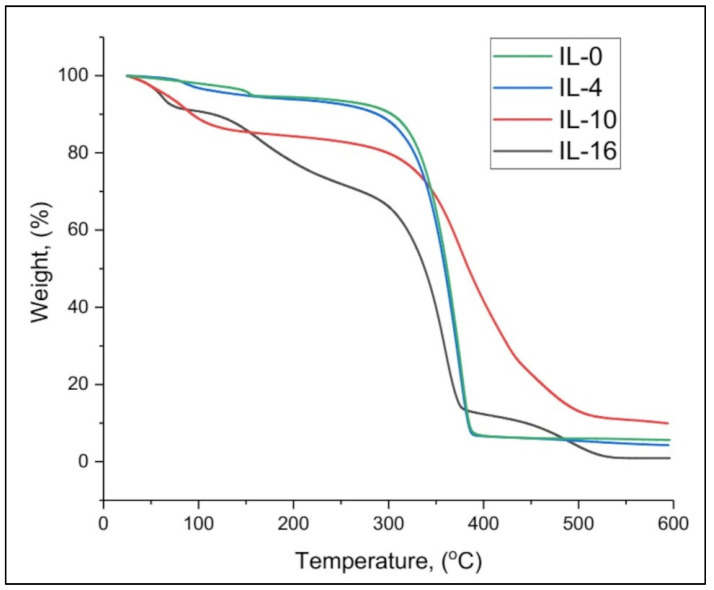
TGA thermograms for the prepared ILs.

**Figure 4:**
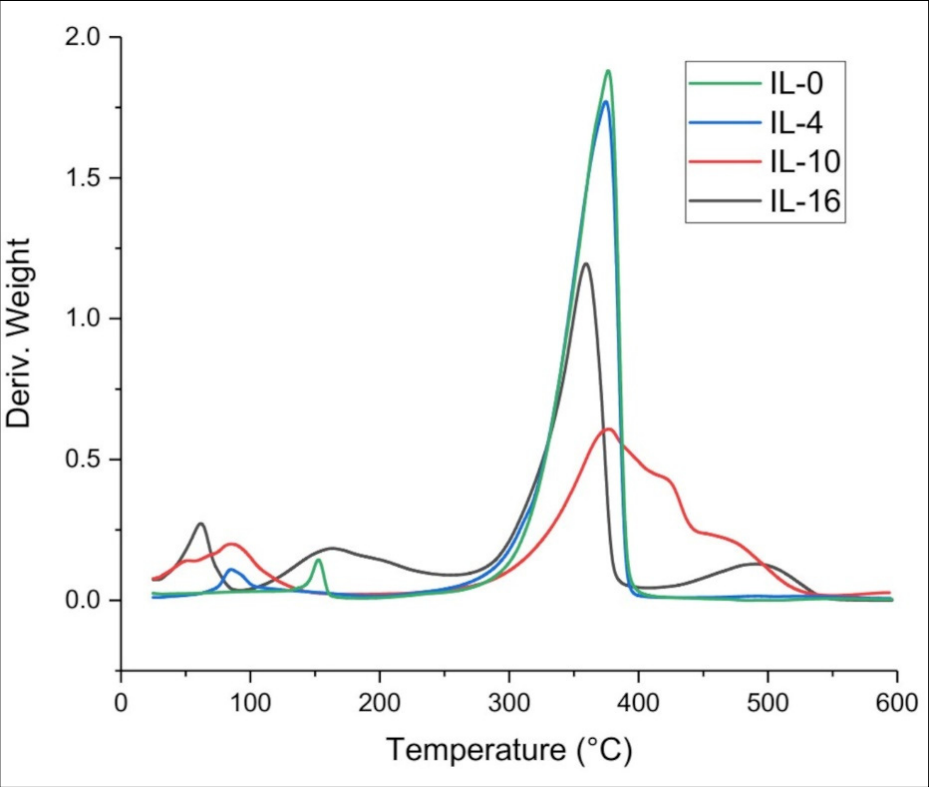
Derivative of TGA thermograms for the prepared ILs.

TGA and DTG composite pictures of IL-0, IL-4, IL-10, and IL-16 are shown in Figure 3 and Figure 4, and the important measurements data are recorded in Table 7. The results confirmed that all of the studied ILs are thermally quite resistant, while IL-0 is the most stable one of them all, followed by IL-4, IL-10, and IL-16. This may be due to the length difference of the alkyl chain attached to the cations of the synthesized ILs. Clearly, the compound with a shorter alkyl chain showed higher thermal stability [57,58].

### 3.6. Quantum Studies

Quantum chemical studies using Density Functional Theory (DFT) were made to investigate the geometry optimization electronic structures, energy gap (ΔE), and reactivity of the cations of the prepared ILs. The quantum chemical calculations were performed by utilizing the basis set 6–31G’(d,p), where Beck’s three-parameter exchange functional, along with the Lee–Yang–Parr nonlocal correlation function (B3LYP), was used.

**Figure 5:**
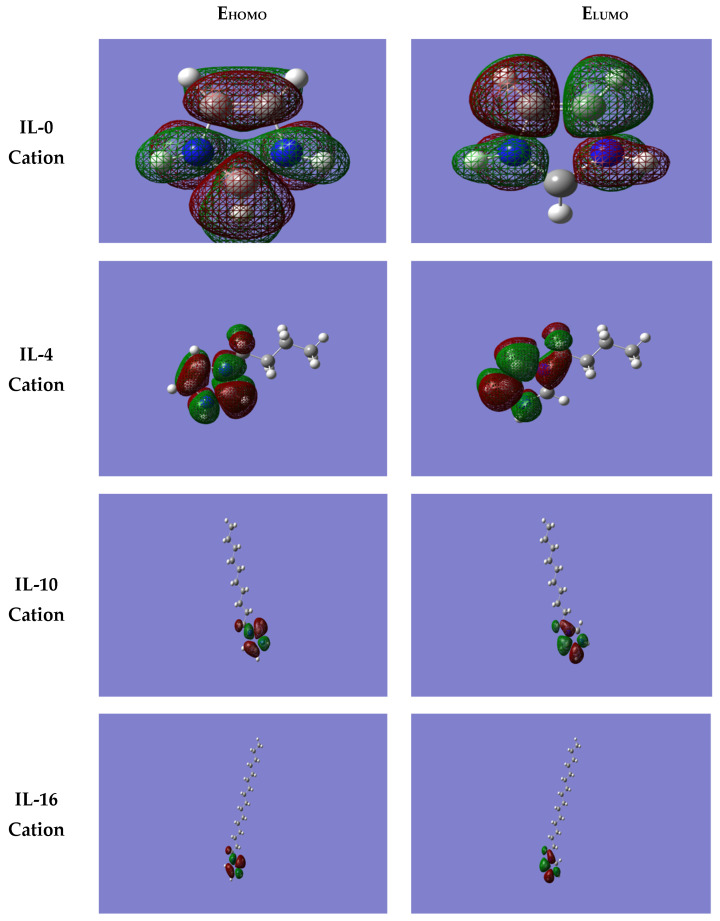
Quantum chemical studies using Density Functional Theory (DFT).

As seen in Table 8, the energy of the highest-occupied molecular orbital (E_HOMO_), the energy of lowest-unoccupied molecular orbital (E_LUMO_), and softness (σ) increased by increasing the alkyl chain length for IL-0, IL-4, IL-10, and IL-16, while the energy gap (ΔE) between E_HOMO_ and E_HOMO_, electrophilicity (ω), hardness (ɳ), and the dipole moment (µ) gradually decreased. The higher value of E_HOMO_ in the order IL-16 < 1L-10 < IL-4 < IL-0 (Figure 5) indicates the tendency of the donor compound to donate electrons to the asphaltene molecules. The reactivity of compounds toward the dispersion of asphaltene molecules is measured by ΔE, which is the difference between E_LUMO_ and E_HOMO_, as shown in Table 8. Increasing the reactivity of the prepared compounds is related to the lowest energy needed to remove electrons from the highest-occupied molecular orbital [59]. The lower ionization energy (I) value of IL-16 indicates the higher dispersion potential toward asphaltene molecules. The higher electronegativity is accompanied by the low reactivity of dispersants. Thus, IL-0 has the highest value of electronegativity (0.01223 ev) and the lowest dispersion activity among the investigated compounds. It is well known that the dipole moment (µ) reflects the global polarity of molecules [60]. Generally, molecules with higher values are more reactive. Thus, IL-16 has a higher (µ) than IL-10 and IL-4. Soft molecules are more reactive than hard ones [61]. IL-16 is the softest IL, while IL-0 is the hardest. Therefore, IL-16 and IL-10 are the preferred asphaltene dispersants, over IL-4 and IL-0.

### 3.7. Surface Tension Measurements of the Prepared ILs

**Figure 6:**
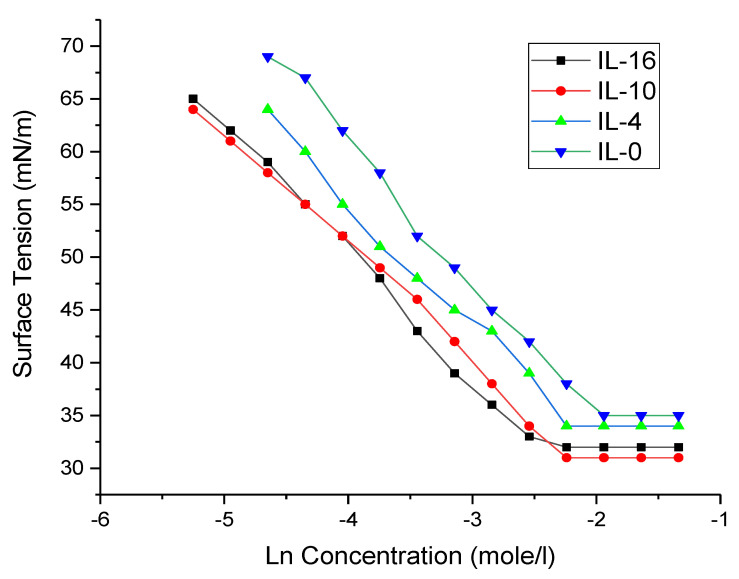
The cmc values of IL-0, IL-4, IL-10, and IL-16.

It is important to investigate the surface-active properties of any chemical while dealing with asphaltenic crude oil, especially at the point of asphaltene precipitation. Thus, surface tension values (γ), critical micelle concentration (cmc), maximum surface pressure (*π*_cmc_), maximum surface excess concentration at surface saturation (Γ_max_), and Gibbs Free Energy for micellization and adsorption of the prepared ILs (IL-0, IL-4, IL-10, and IL-16) were investigated at different temperatures (Table 9). The surface tension values of the prepared ILs were plotted against the logarithm of the corresponding concentrations, as shown in Figure 6. By increasing the concentration of the ILs solution, the surface tension decreases until it reaches a breakpoint known as cmc, after which a plateau is achieved, with no change in the surface tension [62]. The synthesized ILs at the cmc values start to form micelles to decrease the electrostatic energies of the system. The cmc values of the prepared ILs are noted to be lower than of that of other surfactants having similar alkyl chains. Figure 6 shows that increasing the temperature decreases the cmc value of the prepared ILs. This may be due to the reduction in hydration of the hydrophilic part, which favors micellization. It is well known that the most efficient surfactant is that which has the lowest cmc value and the highest π_cmc_ (π_cmc_ = γ − γ), where γ˳ is the surface tension of the water, and γ is the surface tension of the prepared IL, at the cmc value.

From Table 9, IL-10 has the highest π_cmc_ and the lowest cmc and is considered the most effective IL, and the effectiveness increases by increasing the temperature for the synthesized ILs [52]. At the same concentration, the length of the alkyl chains of the four ILs under study has a significant effect on the surface tension reduction. Increasing the hydrophobic chain to 10 or 16 methylene groups exhibits reduced surface tension. This is due to the longer hydrophobic chains having a higher adsorption tendency at the air/water interface. Table 9 shows that IL-10 has a cmc value lower than IL-16; this may be due to the coiling of the long chains. This significant effect rapidly loses its relevance as chain length reaches 16 carbons, and no further appreciable effect of cmc is detected.

#### 3.7.1. The Efficiency of PC20

For any surfactant, the minimum concentration needed to saturate the air/water interface is known as C20 and can be obtained from the curve of γ versus log C. The PC20 values of the prepared ILs showed that IL-10 and IL-16 have the highest values of adsorption efficiency. This reflects that IL-10 and IL-16 have great efficiency in reducing the surface tension of water and show high surface activity.

#### 3.7.2. Maximum Surface Excess Concentration (Γ_max_)

Any material that can lower the surface energy is found at or near the solution’s surface. The max surface excess (Γ_max_) can be calculated from Gibb’s equation as Γ_max_ = (−1/2.303 RT) (d γ/d log C)_T_, where R is the gas const., T is temperature in kelvin, dγ is the surface pressure, and C is the solution concentration of the prepared ILs in mole/l. Table 9 shows the calculated values of Γ_max_ for the prepared ILs, where increasing the temperature from 303 to 323 k decreases the Γ_max_. In addition, the hydrophobic alkyl chain length has an inverse effect on the Γ_max_ [63].

#### 3.7.3. Minimum Surface Area (A_min_)

A_min_ is the minimum area that can be occupied by the surfactant molecule at the interface. It can be calculated from the following equation:

A_min_ = 10^16^/N_A_·Γ_max_,

where N_A_ is Avogadro’s number.

The values of A_min_ listed in Table 9 reveal that the alkyl chain length is directly proportional to the A_min_ values. Additionally, the temperature has the same effect as the alkyl chain, so IL-16 has the highest A_min_ value.

#### 3.7.4. Standard Free Energy of Micellization (ΔG^o^_mic_) and Standard Free Energy of Adsorption (∆G^o^_ads_)

The standard free energy of micellization (ΔG^o^_mic_) is the energy involved in the transfer of one mole of the surfactant from the solution to the micellar phase. At the same time, the adsorption energy (ΔG^o^_ads_) is the required energy to transfer one mole of surfactant from the bulk solution to the interface [2]. The values of both the energies of the prepared ILs can be obtained from the following equations:

ΔG^o^_mic_ = RT ln CMC,

ΔG^o^_ads_ = ΔG^o^_mic_ − (0.6 × π_cmc_ × A_min_).

From the data in Table 9, the values of ΔG^o^_mic_ and ΔG^o^_ads_ are always negative. This points to the fact that both adsorption and micellization processes occur spontaneously, indicating that the processes are thermodynamically favored. This may be due to the repulsion forces between the hydrophobic alkyl chain and the polar solvent. Increasing the alkyl chain length of the prepared ILs increases the negative values of both ΔG^o^_mic_ and ΔG^o^_ads_. Additionally, it can be noted that the values of ΔG^o^_ads_ are larger than those of ΔG^o^_mic_, indicating more spontaneous adsorption processes at the water/air interface than the micellization process in the bulk of the solution [64]. Table 9 shows an increase in the negative values of ΔG^o^_ads_, which promotes the idea of micellization over adsorption at the water/air interface to overcome the resultant repulsion forces. The negative values of free energy of both adsorption and micellization increased with the increase in temperature from 303 to 323 K due to the stability of the adsorption and micellization processes of the ILs solutions.

#### 3.7.5. Interfacial Tension

Table 10 shows the interfacial values of the prepared ILs (IL-0, IL-4, IL-10, and IL-16), which are 10, 7, 3, and 4, respectively. These values are a good indicator of the high efficiency of the prepared ILs at the solution interface [2,53]. Table 10 reveals that IL-10 has the lowest interfacial tension value, while IL-0 has the highest one. These data are in good agreement with the surface tension data.

### 3.8. Evaluation of the Prepared ILs as Asphaltene Dispersants

The prepared ILs (IL-0, IL-4, IL-10, and IL-16) were tested to investigate their effect on the asphaltene precipitation using the viscometric method and UV-vis spectroscopic method [2]. Any effective dispersant or inhibitor can interact or bind with asphaltene functional groups. The selection criteria of imidazolium ILs are based on its structure’s properties that are similar to the properties of resin [65]. They are polar aromatic compounds having long alkyl chains with strong electron donor–electron acceptor properties. The polar aromatic head can interact with polynuclear aromatic moieties in the asphaltene molecules via different types of interactions, such as hydrogen bonding, π–π* stacking, or van der Waal’s force, while the alkyl chain can diffuse in the remaining oil fractions, such as saturates and naphthenes. According to previous studies, the optimum length of the linear alkyl chain attached to the asphaltene dispersant should not exceed 16 carbon atoms. This is may be due to the longer alkyl chain having the ability to crystallize and behave similarly to wax. It is reported that with the addition of enough concentration of an asphaltene dispersant to asphaltene containing oil, the asphaltene becomes soluble in n-heptane. The prepared ILs have good solubility in crude oil; in addition, they can dissolve the asphaltene molecules in the oil system. The amount of dispersant should be sufficient for the amount of asphaltene functional groups. Thus, different concentrations of the ILs (0.1, 0.25, 0.5 wt%) were used to evaluate the optimum concentration.

#### 3.8.1. Viscometric Method

**Figure 7:**
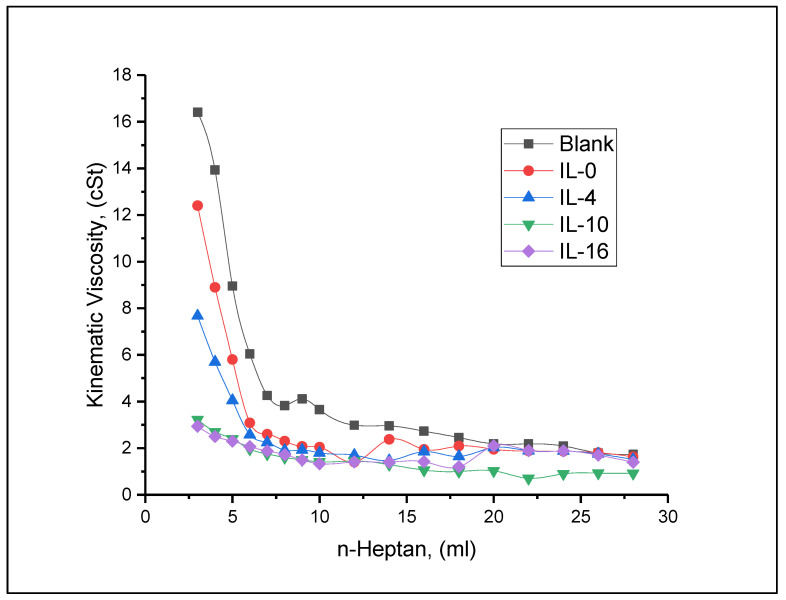
The asphaltene onset precipitation by viscometric method for IL-0, IL-4, IL-10, and IL-16.

Figure 7 shows the effect of n-heptane as the asphaltene precipitant on the kinematic viscosity of the crude oil, with and without adding the prepared ILs (IL-0, IL-4, IL-10, and IL-16), at the same concentration. Increasing the concentration of n-Heptane (up to 80 vol.% of the total mixture) decreased the kinematic viscosity of the treated and untreated oil, as shown in Figure 7. This is due to the dilution effect of the solvent [23]. The asphaltene onset precipitation is the point at the lowest viscosity value, after which a drastic increase in viscosity is observed. According to Figure 7, the asphaltene onset precipitation of the blank crude oil was achieved after adding 28.5 vol.% of n-Heptane. The addition of 0.25 wt% of IL-0, IL-4, IL-10, and IL-16 shifted the onset point to 42.8, 50, 78.5, and 64.3 vol.%, respectively. It was observed that increasing the alkyl chain of the ILs increases the efficiency of dispersion, until they reach the alkyl chain with carbon number 10 (IL-10) [65,66]. However, IL-16 has a higher alkyl carbon chain than IL-10; IL-10 demonstrated a higher efficiency. This may be due to the coiling effect of the extra-long alkyl chain of IL-16. It can be noted that all the ILs delayed the asphaltene onset precipitation to a higher point compared with the natural fatty acids utilized by Reference [24].

**Figure 8:**
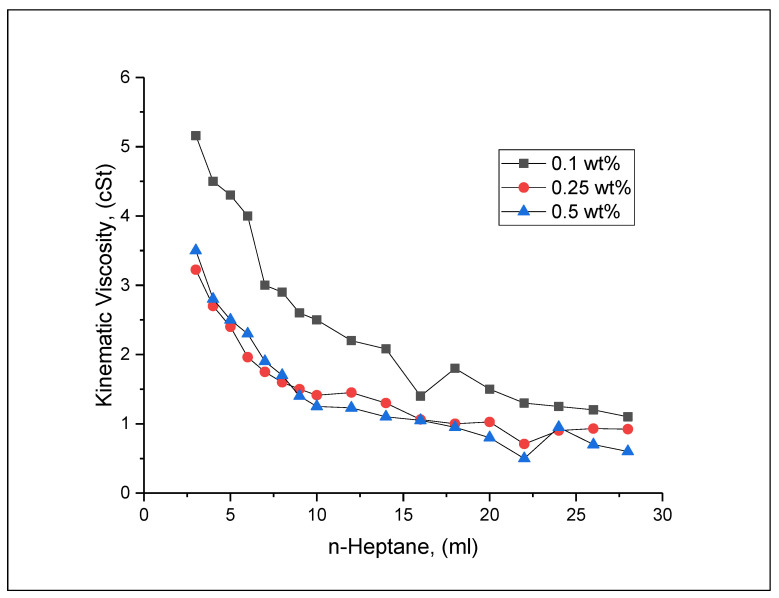
The effect of the concentration of IL-10.

The effect of the concentration of the IL-10 was investigated, and it was noted that increasing the IL concentration increased the asphaltene dispersion power to a certain concentration (0.25 wt.%). Then, increasing the concentration of the IL to 0.5 wt.% had almost the same effect, with no further preference, as shown in Figure 8.

#### 3.8.2. UV-Vis Spectroscopic Method

**Figure 9:**
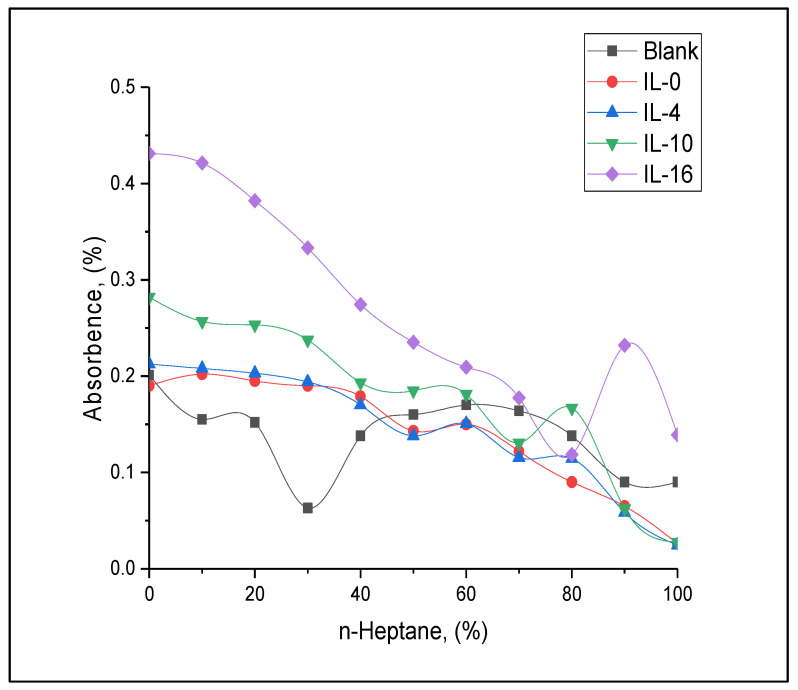
The asphaltene onset precipitation by UV spectroscopic method for IL-0, IL-4, IL-10, and IL-16.

This method was used to assess the effect of the prepared ILs as asphaltene dispersants using a series of n-Heptane/Toluene mixtures (Heptol mixture). The absorbance of Heptol mixtures containing different concentrations of the ILs was detected in the wavelength range of 200–800 nm to determine the asphaltene onset precipitation. The asphaltene onset precipitation was determined from the relation between the absorbance and the concentration of n-Heptane in the Heptol mixture, as shown in Figure 9. It is reported that the asphaltene onset precipitation is represented by the concentration of n-Heptane at the minimum absorbance value [2,66]. The onset of precipitation of the extracted asphaltene in the Heptol mixture was relatively low, at 30% n-Heptane, while it was around 50% for IL-0 and IL-4, and from 70 to 80% for IL-10 and IL-16. These results are in good agreement with the results obtained from the viscometric method. The results reveal that IL-10 and IL-16 are the most effective dispersants, owing to their long alkyl chains and high surface activity. These parameters induce the interaction between the asphaltene molecules and the ILs. Furthermore, they keep the asphaltene molecules colloidally stable in the oil and prevent the formation of aggregates.

It is reported that there is no chemical suitable for all types of crude oils with asphaltene-related issues. However, ionic liquids are the most suitable dispersants because of their good solubility in crude oil and their ability to interact with asphaltenes via different types of interactions [65]. By comparing the efficiency of the prepared ILs with some of the previously prepared compounds, we found that adding 0.25% of IL-0, IL-4, IL-10, and IL-16 postponed the asphaltene precipitation onset from 28.5% to 42.8, 50, 78.5, and 64.3%, respectively. Shadman et al. studied the effects of different compounds as asphaltene dispersants. They found that linear and branched dodecyl benzene sulfonic acid and coconut diethanol amide are good dispersants, as they postponed the asphaltene precipitation onset from 12.2% to 21.2, 18.8, and 20.4%, respectively. When they used triethanolamine lauryl ether sulfate, sodium lauryl ether sulfate, and ethoxylated fatty alcohol 9 mole, the onset point was delayed to only 16.2, 12.2, and 14.6% [65]. Recently, Abdullah et al. presented the effect of different esters of tannic acid as asphaltene dispersants [67]. They stated that the ester of tannic acid increased the needed amount of asphaltene percipient from 60.8% to 75.1%. From the previous results, the efficiency of the prepared ILs compared with the other chemicals can be noted, and we demonstrate that the prepared ILs can be successfully used to control and inhibit the asphaltene molecules in crude oil.

## 4. Conclusions

- Four new ionic liquids were synthesized via the reaction of alkyl imidazoles with dodecylbenzene sulfonic acid. They were characterized well by FT-IR, ^1^H-NMR, and Elemental Analysis.
- The synthesized ILs showed good physico-chemical characteristics, including surface activity and thermal stability.
- The evaluation of ILs as asphaltene dispersants was assessed by spectroscopic and viscometric techniques. The experimental studies indicate that IL-10 and IL-16 are the most effective dispersants, relating to the long alkyl chain and the surface activity.
- Additionally, quantum chemical studies using Density Functional Theory (DFT) were studied to investigate the geometry optimization of electronic structures, energy gap (ΔE), reactivity, hardness, and softness. Increasing the alkyl chain length in the dispersant molecule increased the asphaltene aggregation. Therefore, IL-16 and IL-10 are the preferred asphaltene dispersants, over IL-4 and IL-0.
- All of the previous results (experimental and computational) confirmed the excellent dispersion efficiency of the synthesized ILs (IL-16 and IL-10 more than IL-4 and IL-0).

## Figures and Tables

**Table 1:** Physical properties of the crude oil [53].

Experiment	Method	Result
Density@ 15.56 °C Specific gravity API gravity@15.56 °C	ASTM D-4052	0.9558 0.9568 16.3
Kinematic Viscosity @40 °C, cSt	ASTM D-445	1820.35
Asphaltene content, wt%	IP-143	21.83
Wax content, wt%	UOP-64	1.37
Water content, vol%	ASTM D-97	7.5
Pour point, °C	ASTM D-95	15
Flash point, °C	ASTM D-93	<−22

**Table 2:** Group composition (SARA) analysis of crude oil.

Chemical Composition, wt.%			
Asphaltene	Maltene, wt.%		
		Oil, wt.%	
	Resin	Saturate	Aromatic
21.83	26.52	18.43	33.21

**Table 3:** The chemical structures of the prepared ILs.

ILs	Value	Molecular Structures
**IL-0** **mol wt.** **yield**	394.57 79%	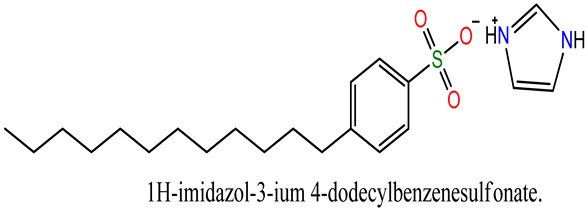
**IL-4** **mol wt.** **yield**	450.68 81%	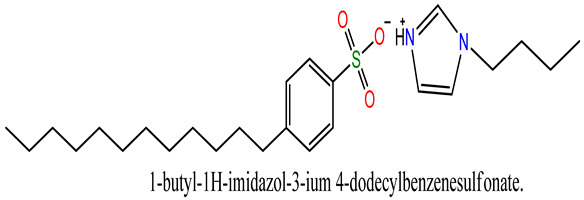
**IL-10** **mol wt.** **yield**	534.84 80%	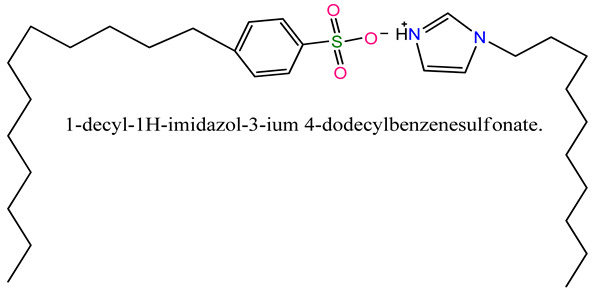
**IL-16** **mol wt.** **yield**	619.00 83%	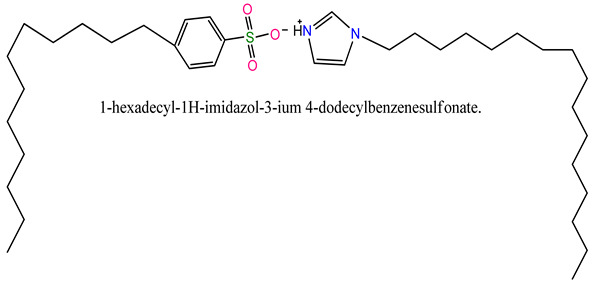

**Table 4:** Elemental analysis of IL-0, IL-4, IL-10, and IL-16.

Elements										
ILs	C%		H%		N%		O%		S%	
	Calc.	Obs.	Calc.	Obs.	Calc.	Obs.	Calc.	Obs.	Calc	Obs.
IL-0	63.92	63.87	8.69	8.73	7.10	7.13	12.16	12.07	8.13	8.20
IL-4	66.63	66.59	9.39	9.42	6.22	6.16	10.65	10.71	7.11	7.12
IL-10	69.62	69.64	10.18	10.15	5.24	5.21	8.97	9.01	6.00	5.99
IL-16	71.79	71.72	10.75	10.81	4.53	4.59	7.75	7.72	5.18	5.16

**Table 5:** FT-IR spectrum of the synthesized ILs.

IL	C-H Aromatic	C-H Aliphatic	C-C Aromatic	C=C Aromatic	C-N	S=O	C-H in Plane Bending	Symmetric Stretching SO_3_^−^
IL-0	3147	2958–2855	1588	1460	1408	1376	1127	1036
IL-4	3137	2958–2855	1579	1462	1407	1377	1126	1034
IL-10	3144	2960–2855	1589	1461	1408	1375	1129	1034
IL-16	3138	2958–2854	1589	1461	1409	1372	1126	1035

**Table 6:** The chemical shift of the prepared ILs (IL-0, IL-4, IL-10, and IL-16).

ILs	Chemical Shift (δ ppm)													
	a	b	c	d	e	f	g	h	i	j	k	l	m	n
IL-0	9.096 (s)	7.81 (d)	7.64 (s)	7.37 (d)	7.13 (d)	2.68 (t)	1.55 (m)	1.05 (t)	0.80 (t)	-	-	-	-	-
IL-4	9.19 (s)	7.8 (d)	7.67 (s)	7.36 (d)	7.25 (d)	7.17 (d)	4.18 (t)	2.63 (m)	1.76 (t)	1.63 (t)	1.36 (m)	1.15 (t)	0.83 (t)	0.75 (t)
IL-10	9.27 (s)	7.80 (d)	7.60 (s)	7.51 (d)	7.13 (d)	2.08 (d)	4.91 (t)	2.54 (t)	1.76 (m)	1.48 (m)	1.21 (m)	0.83 (t)	0.77 (t)	-
IL-16	8.54 (s)	7.61 (d)	7.42 (s)	7.39 (d)	7.12 (d)	7.09 (d)	4.34 (t)	2.67 (t)	1.73 (m)	1.21 (m)	1.13 (m)	0.79 (t)	0.68 (t)	-

**Table 7:** TGA characteristic parameters for the prepared ILs.

Compound	IL-0	IL-4	IL-10	IL-16
**Cation**	1H-imidazole	1-butyl-1H-imidazole	1-decyl-1H-imidazole	1-hexadecyl-1H-imidazole
**First degradation,** **°C**	311	306	303	295
**Final degradation,** **°C**	390	389	490	374

**Table 8:** Quantum chemical properties of the cations of the prepared ILs.

IL’s Cation	E_HOMO_, (eV)	E_LUMO_, (eV)	Energy Gap ΔE, (eV)	Dipole Moment µ, (Debye)	Electron Affinity A, (eV)	Ionization Energy I, (eV)	Electronegativity, (eV mol^−1^)	Hardness ɳ, (eV mol^−1^)	Softness σ, (eV^−1^)
IL-16	−0.04125	0.072	0.11325	0.278734	−0.072	0.04125	−0.015375	0.056625	17.66004
IL-10	−0.04128	0.07201	0.11329	0.278559	−0.07201	0.04128	−0.015365	0.056645	17.65380
IL-4	−0.04159	0.07184	0.11343	0.269447	−0.07184	0.04159	−0.015125	0.056715	17.6320
IL-0	−0.08769	0.06323	0.15092	0.87033	−0.06323	0.08769	0.01223	0.07546	13.25205

**Table 9:** Surface tension measurements of the prepared ILs.

IL	Temperature, °K	CMC mol./L	γ_CMC_, mN/m	π_CMC_ mN/m	Pc_20_	Γ_max_ × 10^−10^ mol./cm^2^	A_min_ nm^2^	ΔG^o^_mic_ KJ/mol	ΔG^o^_ads_ KJ/mol
IL-0	303	1.25 × 10^−2^	35	37.3	3.9 × 10^−4^	2.310	7.1873	−20.412	−22.053
	313	1.13 × 10^−2^	34	38.3	1.757 × 10^−4^	2.016	8.2351	−21.5928	−23.492
	323	1.12 × 10^−2^	33.5	38.8	1.75 × 10^−4^	1.846	8.907	−22.282	−24.383
IL-4	303	6.25 × 10^−3^	34	38.3	1.95 × 10^−4^	1.902	8.725	−23.641	−25.653
	313	5.625 × 10^−3^	33	39.3	8.789 × 10^−4^	1.770	9.377	−24.928	−27.147
	323	2.69 × 10^−3^	33	39.3	8.398 × 10^−4^	1.704	9.738	−29.392	−31.696
IL-10	303	5.75 × 10^−3^	31	41.3	8.98 × 10^−5^	1.880	8.829	−25.977	−28.173
	313	2.75 × 10^−3^	30	42.3	4.29 × 10^−5^	1.681	9.872	−29.692	−32.207
	323	2.63 × 10^−3^	29	43.3	2.05 × 10^−5^	1.609	10.317	−29.926	−32.616
IL-16	303	5.88 × 10^−3^	32	40.3	9.375 × 10^−5^	1.893	8.768	−25.869	−27.997
	313	2.88 × 10^−3^	31	41.3	4.49 × 10^−5^	1.674	9.915	−29.468	−31.934
	323	2.69 × 10^−3^	29	43.3	3.149 × 10^−5^	1.60	10.31	−29.807	−32.498

**Table 10:** Interfacial tension values of the prepared ILs.

IL	Interfacial Tension, mN/m
IL-0	10
IL-4	7
IL-10	3
IL-16	4

## Data Availability

The data that support the findings in the present study are available from the corresponding author upon request.
